# Adenosine and adenosine receptor-mediated action in coronary microcirculation

**DOI:** 10.1007/s00395-021-00859-7

**Published:** 2021-03-23

**Authors:** Ying Zhang, Bernhard Wernly, Xin Cao, S. Jamal Mustafa, Yong Tang, Zhichao Zhou

**Affiliations:** 1 The International Collaborative Centre On Big Science Plan for Purinergic Signalling, Chengdu University of Traditional Chinese Medicine, Chengdu, China; 2 Department of Anaesthesiology, Perioperative Medicine and Intensive Care Medicine, Paracelsus Medical University of Salzburg, Salzburg, Austria; 3 Department of Physiology and Pharmacology, West Virginia University, Morgantown, USA; 4 Acupuncture and Chronobiology Key Laboratory of Sichuan Province, Chengdu, China; 5 Division of Cardiology, Department of Medicine, Karolinska Institutet, Karolinska University Hospital, 17176 Stockholm, Sweden

**Keywords:** Extracellular nucleotides, Purinergic receptor, Coronary microcirculation, Adenosine, Ischemic heart disease, Diabetes

## Abstract

Adenosine is an ubiquitous extracellular signaling molecule and plays a fundamental role in the regulation of coronary microcirculation through activation of adenosine receptors (ARs). Adenosine is regulated by various enzymes and nucleoside transporters for its balance between intra- and extracellular compartments. Adenosine-mediated coronary microvascular tone and reactive hyperemia are through receptors mainly involving A_2A_R activation on both endothelial and smooth muscle cells, but also involving interaction among other ARs. Activation of ARs further stimulates downstream targets of H_2_O_2_, K_ATP_, K_V_ and K_Ca2+_ channels leading to coronary vasodilation. An altered adenosine-ARs signaling in coronary microcirculation has been observed in several cardiovascular diseases including hypertension, diabetes, atherosclerosis and ischemic heart disease. Adenosine as a metabolite and its receptors have been studied for its both therapeutic and diagnostic abilities. The present review summarizes important aspects of adenosine metabolism and AR-mediated actions in the coronary microcirculation.

## Introduction

The coronary microcirculation supplies oxygen and nutrients by determining blood flow to the myocardium through the regulation of vascular resistance. The regulation of coronary microcirculation is essential but complex and is accomplished by changes in coronary microvascular tone, i.e. in contraction and relaxation of vascular smooth muscle, through integration of factors and multiple signals from the perivascular nerves, the myocardium, the endothelium as well as circulating cells [47, 88]. Coronary microvascular dysfunction, resulting in impaired oxygenation and low-grade inflammation, likely contributes to the pathogenesis of coronary microvascular angina [70, 94]. These patients with signs and symptoms of ischemia and non-obstructive coronary artery disease are associated with elevated risk for adverse outcomes [70, 94]. However, the diagnosis of coronary microvascular dysfunction is limited, the disease mechanisms are not fully understood and the patients with non-obstructive coronary artery disease remain under-treated [6, 80].

Adenosine plays a crucial role in the regulation of coronary microvascular tone and coronary blood flow in both physiology and coronary vascular diseases [31, 36, 47]. Adenosine is an autacoid produced by the action of ecto-5′-nucleotidase on extracellular adenine nucleotides released from the parenchymal tissues including endothelium, myocardium and erythrocytes [60, 71]. Extracellular adenosine exerts its vascular effect via interaction with specific cell-surface receptors located on the smooth muscle and endothelial cells of the coronary vasculature. There are four adenosine receptor (AR) subtypes, namely A_1_R, A_2A_R, A_2B_R, and A_3_R. A_1_R and A_3_R are negatively coupled to adenylyl cyclase through the Gi/o protein alpha-subunits and activation of those receptors decreases cAMP levels, whereas A_2A_R and A_2B_R are positively coupled to adenylyl cyclase through Gs and enhance cAMP levels [119]. All four AR subtypes are found in coronary smooth muscle and endothelial cells [3, 31, 67, 76]. The distribution of ARs along the branches of coronary arteries also varies. For instance, in the porcine heart, expression of A_1_R and A_2A_R proteins has been documented in the left anterior descending artery, while A_1_R, A_2A_R and A_2B_R are expressed in coronary arterioles [35, 108]. Despite the fact that the A_2B_R expression is suggested to be restricted to coronary microvascular origin [27, 63], findings from studies using A_2A_R knockout (KO) mice suggested a functional role of A_2B_R in regulating larger coronary arteries than previously thought [96]. The primary effect of adenosine in coronary microcirculation is to induce vasodilation and hyperemia [31, 47]. This property of adenosine to modify coronary microvascular function has been used for diagnostic effects for many years and is widely adopted as the gold-standard method of diagnosing ischemia invasively and noninvasively. The therapeutic potential of adenosine and its ARs has also been studied.

This review summarizes important aspects of adenosine and AR-mediated actions in the coronary microcirculation. The main focus is on the evidence addressing the role of adenosine and involvement of ARs in regulation of coronary microvascular function in physiology. We also discuss the pathophysiology of coronary microvascular regulation in several cardiovascular diseases. Finally, this review briefly touches upon the possible therapeutic potential of adenosine and AR modulation. Considering the differences in heart anatomy and metabolism among different species [89], coronary arteries with the diameter below 200 µm in human and large animal models are included as microvessels in the present review [85], while the changes in flow measured in vivo and ex vivo are regarded as the vasomotor control of the resistance vessels in human, large animal models and rodents.

### Adenosine generation and metabolism

**Fig. 1:**
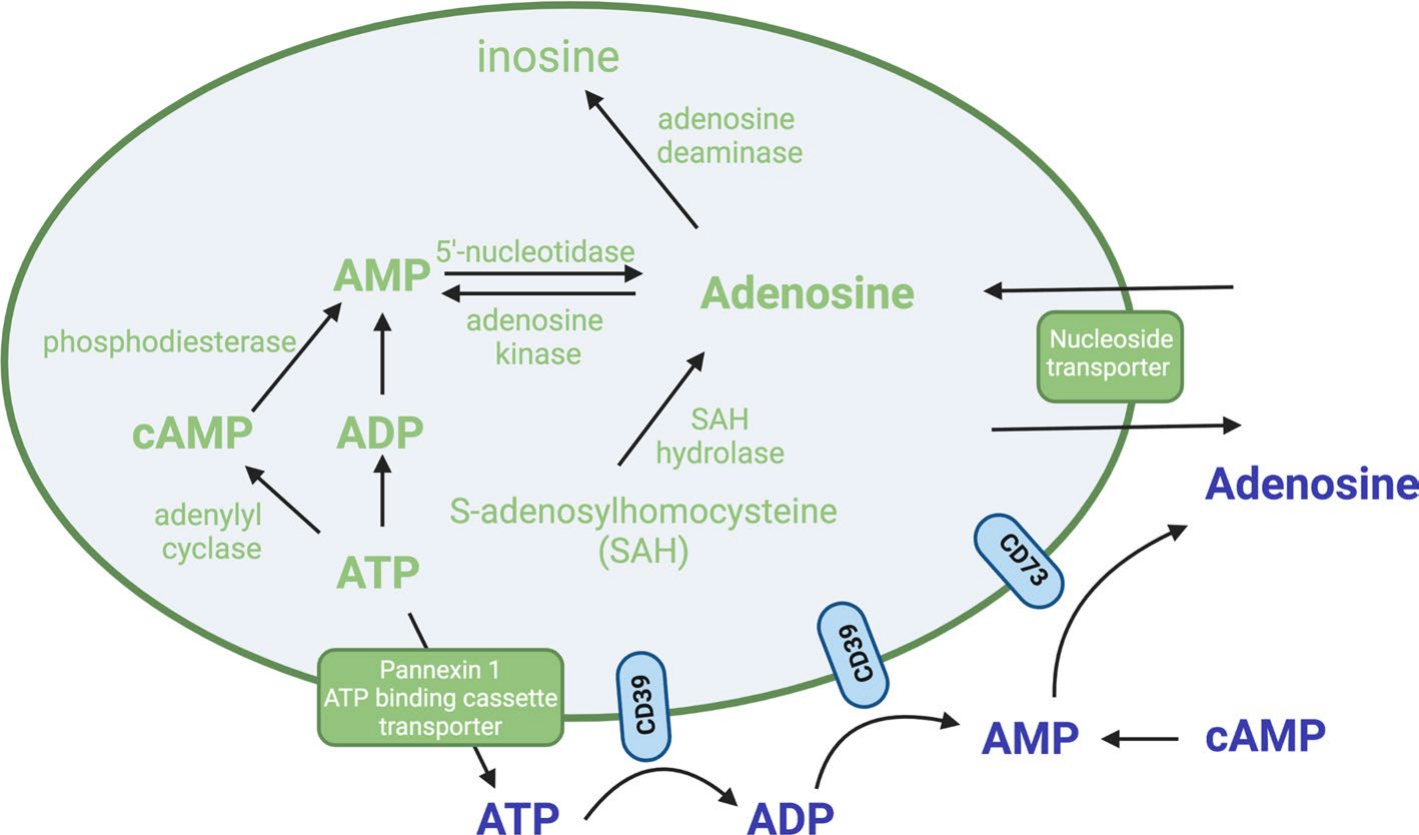
Adenosine generation and metabolism. Adenosine can be formed intracellularly from ATP, ADP or adenosine monophosphate (AMP) by cytoplasmic 5′-nucleosidase activity. The conversion of cAMP to AMP by phosphodiesterase is responsible for adenosine production referring as the cAMP-adenosine pathway. In addition, adenosine can be produced from S-adenosylhomocysteine (SAH) via SAH hydrolase. Once ATP is released extracellularly through pannexin 1 channels or ATP binding cassette transporter, ATP is degraded to ADP and AMP mainly through the continuous action of CD39. Adenosine is then generated from AMP derived from both ATP and cAMP pathways via CD73. Extracellular adenosine is rapidly taken up by the cells via nucleoside transporters for subsequent metabolism. Adenosine is then phosphorylated in the cells by adenosine kinase to form AMP or degraded to inosine by adenosine deaminase

Adenosine is released in coronary microcirculation from tissues including endothelium, myocardium and erythrocytes at times of cellular stress such as hypoxia, ischemia and inflammation [60]. Adenosine can be formed intracellularly from ATP, ADP or adenosine monophosphate (AMP) by cytoplasmic 5′-nucleosidase activity. The conversion of cAMP to AMP by phosphodiesterase (PDE) is responsible for adenosine production referred to as the cAMP-adenosine pathway [73]. In addition, adenosine can be produced from S-adenosylhomocysteine (SAH) via SAH hydrolase [21, 82]. Once being released extracellularly, ATP is degraded to ADP and AMP through the continuous action of NTPDase 1 (CD39) or possibly other NTDPases [119, 124]. Adenosine is then generated from AMP derived from both ATP and cAMP pathways via CD73 [73] (Fig. 1).

Alteration of the regulatory enzyme activity under (patho)physiological conditions or in response to pharmacological stimuli can affect adenosine levels and subsequently the AR-mediated vascular responses. Physical training may increase cytoplasmic 5′-nucleosidase and adenosine deaminase activity, thereby affecting adenosine concentration [46]. 5′-nucleosidase activity was thought to be inhibited during ischemia or hypoxia [29]. However, the net adenosine concentration was not measured. In a setting of reduced tissue oxygenation, the adenosine level can be elevated more than the AMP level likely via decreased activity of adenosine kinase [19]. Upon β-adrenergic stimulation, SAH-hydrolase was inhibited via a calcium-dependent mechanism [90], while CD73 activity was increased [32]. Hypoxia can also increase CD73 activity resulting in increased extracellular cardiac adenosine production [33]. α1-adrenergic stimulation, nitric oxide (NO)-donors and 8-bromo-cGMP could stimulate PKC leading to increased activity of CD73 [4, 65]. Pharmacological inhibition of adenosine deaminase and kinase in perfused mouse hearts resulted in a significant increase in coronary flow [93].

Adenosine can diffuse across cell membranes to maintain the balance between intracellular and extracellular adenosine concentrations. Extracellular adenosine is rapidly taken up by the cells via both sodium-dependent (concentrative nucleoside transporter: CNT) and sodium-independent transporters (equilibrative nucleoside transporter: ENT) for subsequent metabolism [50, 51, 53]. Further, adenosine can pass through the plasma membrane of these cells and be used intracellularly [73]. Once taken up, e.g., by the endothelial cells, adenosine is phosphorylated by adenosine kinase to form AMP or degraded to inosine by adenosine deaminase (ADA) [73] (Fig. 1). Both ENT and CNT are expressed in the heart and vessel [53]. However, ENT1 and ENT2 are the best-characterized transporters for adenosine uptake in the cardiovascular system [50]. Existing evidence has shown that targeting ENT contributes to coronary vasodilation [7, 39]. ENT1 and ENT2 are the predominant nucleoside transporters of the vascular endothelium with an approximate expression of ENT1 twice as high as that of ENT2 [51]. The human ENT1 and ENT2 differ in their sensitivities to inhibition by coronary vasodilators such as dipyridamole, dilazep and draflazine, with ENT1 being ≈100- to 1000-fold more sensitive than ENT2 [104].

However, there are several limitations in our current understanding of adenosine metabolism. The mechanisms underlying the regulation of these enzymes and transporters are not fully understood, which deserves further investigations. In addition, more studies are needed using human tissues, as there are species differences with respect to adenosine metabolism [20]. Finally, how altered enzyme activity and adenosine concentration affects sensitivity and activation of ARs in coronary microcirculation remains poorly elucidated. For more details on adenosine metabolism, the reader is referred to several excellent review articles [20, 81].

### Adenosine-mediated actions in physiological conditions

#### Involvement of ARs in coronary microvascular tone control

**Fig. 2:**
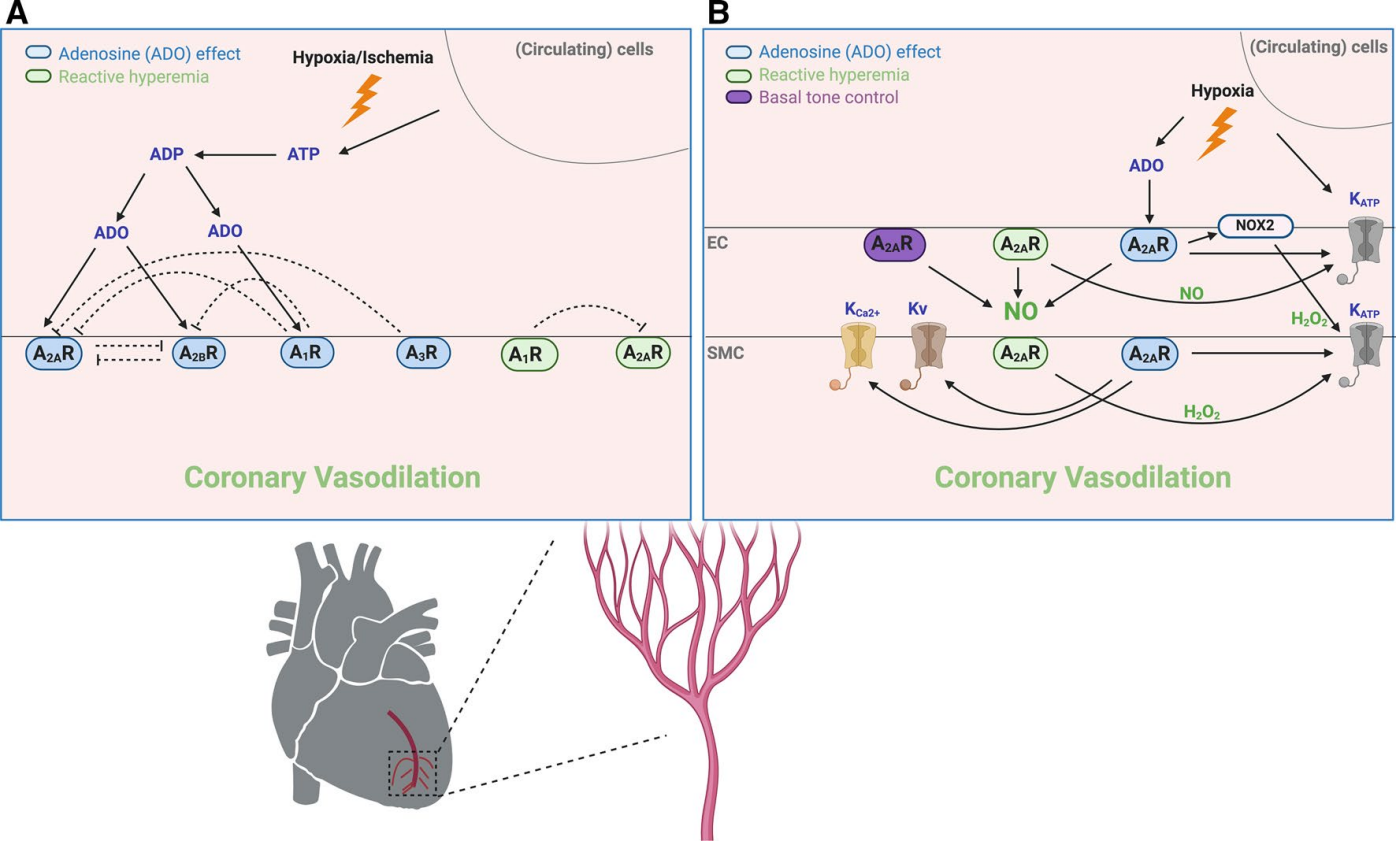
Adenosine and adenosine receptor (AR)-mediated action in coronary microcirculation in physiology. **a** Adenosine is generated via extracellular breakdown of ATP released from various cells upon stimulation like hypoxia or ischemia. Adenosine-mediated coronary microvascular tone is mainly through activation of A_2A_R and A_2B_R. A_2A_R and A_2B_R can compensate for each other, while A_1_R and A_3_R negatively modulate the A_2A_R- and A_2B_R-mediated coronary vasodilation. A_2A_R plays a role in coronary reactive hyperemia. A_1_R negatively modulates coronary reactive hyperemia mediated by A_2A_R. **b** There are endothelium-dependent and -independent regulations of adenosine-mediated coronary microvascular function. Nitric oxide (NO) is involved in A_2A_R-mediated basal tone control and reactive hyperemia, as well as adenosine-mediated A_2A_R activation. NO is also involved in A_2A_R-K_ATP_ axis for reactive hyperemia. Activation of A_2A_R can stimulate NADPH oxidase 2 (NOX_2_) resulting in H_2_O_2_ production, which leads to smooth muscle cell (SMC) K_ATP_ opening and coronary vasodilation. Activation of A_2A_R by reactive hyperemia also involves downstream H_2_O_2_-K_ATP_ axis accounting for coronary vasodilation. Hypoxia can directly activate K_ATP_ channels. Involvement of SMC Kv and K_Ca2+_ is coupled to activation of A_2A_R. *EC* endothelial cells

Adenosine is a potent coronary vasodilator in all species studied, including human [15, 60, 69, 109, 126]. It can arise directly from cardiomyocytes after intracellular breakdown of ATP and after extracellular breakdown of ATP released from endothelial cells and erythrocytes [71]. The involvement of ARs in adenosine-mediated coronary vasodilation is species dependent. Several lines of evidence have shown that both A_2A_R and A_2B_R mediate exogenous adenosine-induced coronary vasodilation in mice [59, 86, 93], while A_2A_R is the predominant receptor subtype contributing to coronary vasodilation in swine and dogs [9, 35, 52, 125]. Involvement of ARs in human coronary vascular tone is not consistent. Activation of A_2A_R has been shown to regulate human coronary vascular tone [79], whereas another study indicates an involvement of A_2B_R in adenosine-induced relaxation in small arteries isolated from human [42]. ARs also interact with each other to regulate coronary vascular tone. Both A_1_R and A_3_R have been found to negatively modulate coronary vasodilation induced by A_2A_R and/or A_2B_R activation [92, 95]. The A_2B_R expression is upregulated in coronary arteries isolated from mice lacking A_2A_R. As a functional consequence, the A_2B_R-mediated increase in coronary flow is enhanced in mice lacking A_2A_R [96]. Further, the A_2A_R-mediated increase in coronary flow is enhanced in mice lacking A_2B_R [78] (Fig. 2). Whether ARs play a significant role in the regulation of coronary basal tone remains controversial. In isolated rat hearts, the coronary baseline flow is significantly reduced by non-selective AR inhibition [49]. A_2A_R activation has been observed to contribute to coronary basal NO release and basal tone in isolated hearts of mice [28, 117, 120] (Fig. 2). In contrast, the effect of AR blockade on coronary blood flow in vivo is rather small in human [25, 26] and swine [24], and even absent in dogs and mice [5, 99, 115].

As mentioned earlier, upon induction of hypoxia or ischemia in various tissues, adenosine together with ATP and ADP is released from cells or tissues, all of which significantly contribute to reactive hyperemia [68, 81]. It has been proposed that adenosine and adenosine-mediated ARs predominantly account for the mid- to late-phase of reactive hyperemia [68]. Existing evidence demonstrates that activation of A_2A_R plays a pivotal role in reactive hyperemia in mice and dogs [9, 86, 117, 122]. Other receptors play a lesser role. For instance, A_1_R has been shown to negatively modulate coronary reactive hyperemia mediated by A_2A_R [122] (Fig. 2). A_2B_R seems not to be involved in coronary reactive hyperemia [86, 122]. There is also evidence showing that adenosine is unlikely to be involved in coronary reactive hyperemia [10, 22].

Adenosine levels (calculated) do not increase enough to reach the concentration threshold to cause coronary vasodilation with increasing exercise intensity in human, swine and dogs [24, 100], and there is no evidence for increased myocardial interstitial levels of adenosine following adenosine receptor blockade [100, 115]. No involvement of adenosine or A_2A_R has also been observed in a mouse model with pacing-induced coronary hyperemia [121]. These findings indicate that adenosine is not mandatory for coronary metabolic hyperemia.

Existing evidence demonstrated divergent effects induced by activation of A_1_R and A_3_R on coronary microvascular function. Vasodilator effect mediated by A_1_R and A_3_R has been evidenced by the A_1_R-induced vasodilation in canine coronary microcirculation [16] and the A_3_R-produced coronary vasodilation in isolated rat hearts [40, 76]. In contrast, A_1_R antagonism augments the sensitivity to adenosine in isolated human coronary arterioles [79]. Further, both A_1_R and A_3_R have been found to negatively modulate coronary vasodilation induced by A_2A_R and/or A_2B_R activation in isolated mouse hearts [92, 95], and A_1_R counteracts the A_2A_R-mediated coronary reactive hyperemia [122] (Fig. 2).

#### Endothelium-dependent and -independent regulation

It has been suggested that both A_2A_R and A_2B_R mediate endothelium-dependent coronary relaxation and NO release from coronary artery endothelium [1]. Indeed, adenosine-5′-N-ethylcarboxamide (NECA), a nonselective adenosine agonist, and 2-[p-(2-carboxyethyl)] phenylethyl-amino-5′-N-ethylcarboxamidoadenosine (CGS-21680), a selective A_2A_R agonist, produced relaxation in isolated porcine coronary small arteries, which were attenuated by the endothelium-denudation or NO synthase inhibition [34]. Using two different NO synthase inhibitors L-NAME and L-NMA in isolated hearts from wild-type (WT) and A_2A_R KO mice, both inhibitors attenuated the NECA- or CGS-21680-induced increases in coronary flow in WT, but not A_2A_R KO mice, indicating a role for NO in the A_2A_R-mediated coronary vasodilation [96]. NO blockade or endothelium denudation also attenuated adenosine-induced vasodilation in porcine coronary arterioles [44]. Interestingly, adenosine-A_2A_R pathway has been shown to regulate coronary basal tone through NO release in isolated mouse hearts [120]. There is also evidence showing that NO release is in part triggered by A_2A_R accounting for reactive hyperemia in mice [117]. The role of A_2B_R in NO release remains to be determined.

In contrast, many other studies have observed that adenosine mediates endothelium-independent relaxation in coronary microvasculature. Thus, adenosine-induced vasodilation in human coronary small arteries was not affected by endothelium denudation [79] or NO blockade [42]. NO synthase inhibition failed to affect adenosine-induced vasodilation in canine coronary arterioles [41]. Endogenous adenosine and NO work in a parallel manner to regulate vascular tone in isolated canine coronary small arteries [116]. NO does not contribute to the A_2A_R-mediated increase in reactive hyperemia in A_1_R KO mice [122]. Numerous pieces of evidence obtained in denuded porcine coronary small arteries clearly demonstrated that A_2A_R plays a predominant role in endothelial-independent coronary vasodilation, while A_2B_R may play a minor role [91, 98, 125]. The discrepancies on the role of endothelium in the adenosine-mediated coronary microvascular regulation are not readily explained, but may be determined by the different expression and distribution of ARs between the endothelium and smooth muscle cells in the different vascular segments of the microcirculation. It may also depend on different species studied, as NO seems to be involved in adenosine-induced coronary vasodilation in swine, but not dogs [41]. Further studies are warranted to address this issue.

#### Post-receptor pathways and end-effectors

The coronary microvascular tone is ultimately determined by the interaction between actin and myosin in the vascular smooth muscle cells. This is regulated by the intracellular Ca^2+^ concentration. Opening status of one of the important modes voltage-operated Ca^2+^ channels in vascular smooth muscle is regulated by membrane potential, which in turn is determined by the activation of K^+^ channels [24]. Many vasoactive substances including H_2_O_2_ influence coronary microvascular function through K^+^ channels [64, 75, 78, 86, 118]. The limited evidence regarding the mechanisms is pointed to a possible activation of both transcription and translation of K^+^ channels located at the plasma membrane of the coronary smooth muscle cells [64]. The three main types of K^+^ channels that have been investigated in relation to regulation of coronary vasomotor tone are K_ATP_, K_Ca2+_ and K_V_ channels [23]. Despite information indicating that adenosine receptors and K_ATP_ work as parallel vasodilator pathways to control coronary blood flow in swine [56], both A_2A_R- and A_2B_R-mediated increase in coronary flow in isolated mouse hearts have been observed to be through activation of K_ATP_ channels [78]. The adenosine/A_2A_R stimulation- or the adenosine analogue-induced relaxation in isolated porcine coronary arterioles or the A_2A_R-induced increase in coronary blood flow in open-chest dogs is mediated via activation of K_ATP_ channels [8, 9, 34]. Adenosine has been shown to potentiate the flow-mediated dilation in porcine coronary arterioles via activation of K_ATP_ channels in endothelium [44]. There is NO and K_ATP_ channel-dependent effects of A_2A_R contributing to reactive hyperemia in mouse [117]. Of further interest, recent evidence has shown that A_2A_R activation promotes NADPH oxidase 2-derived reactive oxygen species and subsequently leads to H_2_O_2_ production contributing to the increase in coronary flow in isolated mouse hearts [126]. The interaction between A_2A_R, H_2_O_2_ and K_ATP_ has been demonstrated in delicate models of A_2A_R KO and A_2A_R/A_2B_R double KO mice. Thus, patch-clamp experiments demonstrated that adenosine can activate glibenclamide-sensitive K_ATP_ current in smooth muscle cells from WT, but not A_2A_R KO or A_2A_R/A_2B_R double KO mice [86]. H_2_O_2_ can activate K_ATP_ current in smooth muscle cells [86]. Further, adenosine-mediated increase in coronary flow is blunted by catalase, while H_2_O_2_ increases coronary flow which is attenuated by the K_ATP_ blocker glibenclamide [86]. Finally, both H_2_O_2_ and K_ATP_ activation are involved in A_2A_R-mediated coronary reactive hyperemia [86, 122]. Altogether, these observations indicate that adenosine-mediated A_2A_R is coupled to smooth muscle K_ATP_ channels in coronary reactive hyperemia in mice via the production of H_2_O_2_ as a signaling intermediate. Earlier studies have also shown an involvement of K_ATP_ channels in hypoxia-induced coronary vasodilation as well as dipyridamole-mediated increase in coronary vasodilation in perfused guinea pig hearts [18, 106], suggesting that adenosine could hyperpolarize smooth muscle cell membrane by opening K_ATP_ channels under hypoxic condition.

There is also evidence showing the involvement of K_V_ and K_Ca2+_ channels in adenosine- or the A_2A_R agonist-mediated coronary vasodilation. Adenosine-mediated increases in coronary blood flow in dogs and relaxation in isolated canine coronary arteries, the adenosine analogue-induced relaxation in isolated porcine coronary arterioles, as well as the adenosine/the A_2A_R agonist-induced relaxation in coronary arteries isolated from rats are attenuated by K_V_ channel inhibition [8, 9, 22, 43]. Moreover, adenosine-mediated vasodilation in pressurized human and canine coronary small arteries and in perfused rat hearts were blunted by K_Ca2+_ channel inhibition [11, 58, 79].

Collectively, adenosine-mediated coronary microvascular tone and reactive hyperemia are through complex mechanisms mainly involving A_2A_R activation on both endothelial and smooth muscle cells, but also involving the interaction of different ARs (Fig. 2). Regarding the post-receptor mechanism, K_ATP_, K_V_ and K_Ca2+_ channels appear to act as final effectors for the adenosine-mediated coronary microvascular tone regulation. However, the mechanisms underlying how adenosine or the A_2A_R-mediated coronary vasodilation activates H_2_O_2_-K_ATP_ axis remains incompletely understood. Moreover, whether adenosine-H_2_O_2_-K_ATP_ axis can be extrapolated to human condition deserves further investigations. Table 1 summarizes important evidence regarding the role of adenosine- and AR-mediated actions in coronary microcirculation in physiology.

**Table 1:** Adenosine and adenosine receptor-mediated coronary microvascular function in physiology

Species	Age/weight, sex	Coronary function assessement method	Receptor/pathways	Coronary effect	Reference
Human	Not stated	Isolated coronary arterioles (internal diameter 0.4 μm) in pressurized myograph	A_1_R, A_2A_R, IKCa^2+^	↓ Ado-mediated vasodilation + DMPX or Clotrimazole ↓ Ado-mediated vasodilation + DPCPX - Ado-induced vasodilation + endothelium denudation	[79]
Human	64.4 ± 1.7 years, either sex	Isolated coronary small arteries (diameter: ~ 200 μm) in wire myograph	A_2B_R	↓ Ado-induced relaxation + DMPX -Ado-induced relaxation + LNAME or Glibenclamide	[42]
Swine	8–12 weeks, either sex	Isolated coronary arterioles (diameter 50–100 μm) in pressurized myograph	A_2A_R, K_ATP_	↓ Ado and CGS21680-mediated vasodilation + ZM241385 or Glibenclamide	[35]
Swine	8–12 weeks, either sex	Isolated coronary arterioles (diameter 50–100 μm) in pressurized myograph	A_2A_R, NO, K_ATP_	↑ Ado, NECA, ENBA, CGS21680, IB-MECA-induced vasodilation ↓ Ado-induced vasodilation + ZM241385 but not CPX and MRS1191 ↓ Ado and CGS21680-induced vasodilation + LNAME or endothelium denudation ↓ Ado and CGS21680-induced vasodilation in denuded vessel + Glibenclamide	[34]
Swine	8–12 weeks, either sex	Isolated coronary arterioles (diameter 50–100 μm) in pressurized myograph	NO, K_ATP_	↓ Ado-potentiated flow-induced vasodilation + Glibenclamide ↓ Ado-induced vasodilation + LNAME or endothelium denudation	[44]
Swine	2–3 months, either sex	Catherization in the anterior interventricular vein	ARs	↓↓ P_VO2_ + 8PT + Glibenclamide or 8PT + LNAME vs. 8PT	[56]
Miniature swine	9–12 months, male	Isolated coronary arterioles (diameter: 50–150 μm) in pressurized myograph	A_2A_R, A_2B_R, Kv, K_ATP_	↓ 2-CAD-induced vasodilation + ZM241385 or 4AP or Clibenclamide	[8]
Miniature swine	14 ± 4 months	in vivo intravascular ultrasound	A_2A_R	↓ Ado-mediated increase in CBF + ZM241385	[52]
Dog	4–11 kg, either sex	Isolated coronary arterioles (diameter ~ 81 μm) in pressurized myograph		- Ado-induced vasodilation + LNAME	[41]
Dog	10–25 kg, either sex	Isolated coronary arterioles (diameter ~ 100 μm) in pressurized myograph	ARs	↓↓ Coronary vasodilation with LNAME + Catalase + 8PT vs. LNAME + Catalase	[116]
Dog	Not stated	Isolated coronary arterioles (diameter ~ 170 μm) in pressurized myograph	ARs, BKCa^2+^	↓ Ado-induced vasodilation + TEA or iberiotoxin	[11]
Dog	20–30 kg, male	CBF measurement in open-chest dog	A_2A_R, A_2B_R, Kv, K_ATP_	↓ Ado-induced increase in CBF + SCH58261 or Alloxazine ↓ CGS21680-mediated increase in CBF + 4AP or Glibenclamide ↓ RH-induced increase in CBF + SCH58261	[9]
Dog	Not stated	CBF measurement in open-chest dog, isolated arterioles (diameter: ~ 150 μm) in pressurized myograph	AR, Kv	↓ RH-induced increase in CBF with 8PT + 4AP but not 8PT ↓ Ado-induced increase in CBF in vivo and relaxation ex vivo + 4AP	[22]
Dog	Not stated	CBF measurement in open-chest dog	A_1_R, A_2A_R	↑ CCPA and DPMA-induced increase in CBF	[16]
Rat	11–16 weeks, male	Isolated coronary small arteries (diameter: ~ 200 μm) in wire myograph	A_2A_R, Kv_7_	Ado and CGS21680-induced relaxation + linopirdine	[43]
Rat	280–380 g, male	ex vivo perfused hearts Langendorff technique	A_2_R	↓ Baseline CF + 8PT	[49]
Rat	6–8 weeks and 16–18 weeks and 52–54 weeks, male	ex vivo perfused hearts Langendorff technique	A_3A_R	↓ APNEA-induced increase in CF + MRS1191 or Alloxazine	[40]
Rat	10–12 and 18–20 weeks	ex vivo perfused hearts Langendorff technique	ARs, IKCa^2+^, SKCa^2+^	Ado-induced increase in CF + TRAM34 or TRAM34 + Apamin	[58]
Guinea pig	350–450 g, male	ex vivo perfused hearts Langendorff technique	A_1_R, A_2A_R, A_3_R	↑ ADAC, CCPA and APNEA-induced decrease in perfusion pressure	[76]
Guinea pig	200–300 g	ex vivo perfused hearts Langendorff technique	ARs, K_ATP_	↓ Hypoxia-induced vasodilation + Glibenclamide ↓ Dipyridamole-induced vasodilation + 8PT	[106]
Mouse	Adult male and female	ex vivo perfused hearts Langendorff technique	A_2A_R, A_2B_R	↓ Ado or NECA-mediated increase in CF in A_2A_R KO mice ↓ NECA-mediated increase in CF + Alloxazine	[59]
Mouse	10–14 weeks, male	ex vivo perfused hearts Langendorff technique	A_2A_R, H_2_O_2_, NO, K_ATP_	↓ RH-induced increase in CF in A_2A_R or A_2A/A2B_R KO mice ↓ RH-induced increase in CF in A_2A_R or A_2A/A2B_R KO mice + Glibenclamide ↓ Ado-induced increase in CF in WT mice + Catalase ↓ Ado-induced K_ATP_ current in SMCs of A_2A_R KO mice ↓ H_2_O_2_-induced K_ATP_ current in SMCs + Glibenclamide ↓ RH-induced increase in CF + Catalase in WT but not A_2A_R KO ↓↓ RH-induced increase in CF + LNAME + Catalase vs. LNAME in WT	[86]
Mouse	Adult male and female	ex vivo perfused hearts Langendorff technique	A_2A_R, A_2B_R	↓ EHNA and ITU-induced increase in CF in A_2A_R KO mic ↓ EHNA and ITU-induced increase in CF in A_2A_R KO + Alloxazine	[93]
Mouse	Adult male and female	ex vivo perfused hearts Langendorff technique	A_2A_R, A_3_R	↑↑ Adenosine and CGS21680-induced increase in CF in A_3_R KO vs. WT mice	[92]
Mouse	Adult male and female	ex vivo perfused hearts Langendorff technique	A_1_R, A_2A_R	↑ Basal CF in A_1_R KO vs. WT mice ↑ Adenosine and CGS21680-induced increase in CF in A_1_R KO vs. WT mice ↓ Adenosine and CGS21680-induced increase in CF in WT mice + DPCPX	[95]
Mouse	Adult male and female	ex vivo perfused hearts Langendorff technique	A_2A_R, A_2B_R, NO	↓ NECA-induced increase in CF in WT but not A_2A_R KO mice + LNAME ↓ CGS21680-induced increase in CF in WT mice + LNAME ↑ BAY606583-mediated increase in CF in A_2A_R KO vs. WT mice	[96]
Mouse	10–14 weeks, male	ex vivo perfused hearts Langendorff technique	A_2A_R, A_2B_R, K_ATP_	↓ NECA-induced increase in CF in A_2B_R KO mice + SCH58261 ↓↓ NECA-induced increase in CF in A_2A/2B_R KO mice ↑ CGS21680-induced increase in CF in A_2B_R KO vs. WT mice ↓ NECA-indcuced increase in CF in WT, A_2A_R and A_2B_R KO mice + Glibenclamide ↓ CGS21680 and BAY605683-induced increase in CF in WT mice + Glibenclamide	[78]
Mouse	12–16 weeks, either sex	ex vivo perfused hearts Langendorff technique	A_1_R, A_2A_R, H_2_O_2_, K_ATP_	↑ RH-induced increase in CF in A_1_R KO and A_1/3_R KO vs. A_3_R KO mice ↓ RH-induced increase in CF in A_1_R KO mice + SCH58261 or Catalase or Glibenclamide but not LNAME	[122]
Mouse	14–18 weeks, either sex	ex vivo perfused hearts Langendorff technique	A_2A_R, NOX_2_, H_2_O_2_	↓ Ado and CGS21680-induced increase in CF in WT, A_2B_R but not A_2A_R KO mice + gp91 ds-tat or EUK134 ↓ Ado-induced increase in H_2_O_2_ formation in WT, A_2B_R but not A_2A_R KO mice + gp91 ds-tat	[126]
Mouse	7–12 weeks	ex vivo perfused hearts Langendorff technique	A_2_R	↓ Baseline CF + 8-CSC	[28]
Mouse	8–12 weeks, male	ex vivo perfused hearts Langendorff technique	A_2A_R, NO, K_ATP_	↓ Baseline CF + SCH58261 ↓ RH-induced increase in CF + SCH58261 or LNAME or Glibenclamide	[117]
Mouse	20–22 weeks, either sex	ex vivo perfused hearts Langendorff technique	A_2A_R	↓ Baseline CF in WT mice + SCH58261 ↓↓ RH-induced increase in CF in ApoE KO + HFD vs. WT mice + SCH58261	[120]
Mouse	Adult male and female	In vivo ultrasound CBF measurement	A_2A_R, A_2B_R	↓ i.v. bolus Ado-induced increase in CBF in A_2A_R, A_2B_R, and A_2A/2B_R KO mice	[99]

AR agonist: Adenosine (Ado), NECA; A_1_R agonist: ENBA, CCPA, ADAC; A_2A_R agonist: CGS21680, DPMA; A_2B_R agonist: 2-CAD, BAY606583; A_3_R agonist: APNEA, Cl-IB-MECA; AR antagonist: DMPX, 8PT; A_1_R antagonist: DPCPX, CPX; A_2_R antagonist: 8-CSC; A_2A_R antagonist: ZM241385, SCH58261; A2BR antagonist: Alloxazine; A3R antagonist: MRS1191; ApoE: Apolipoprotein E; Adenosine deaminase inhibitor: EHNA; Adenosine kinase inhibitor: ITU; Big conductance calcium-activated potassium channel blocker: iberiotoxin; Calcium-activated potassium channel blocker: Clotrimazole; *CBF* coronary blood flow; *CF* coronary flow; *HFD* high fat diet; *H*_*2*_*O*_*2*_ decomposition catalyst: catalase; Intermediate conductance calcium-activated potassium channel blocker: TRAM34; K_ATP_ channel blocker: Glibenclamide; Kv channel blocker: 4AP; Kv7 channel blocker: linopirdine; NADPH oxidase 2 inhibitor: gp91 ds-tat; Non-selective potassium blocker: TEA; Nitric oxide (NO) synthase inhibitor: LNAME; PvO_2_: coronary venous O_2_ pressure; Reactive oxygen species (ROS) scavenger: EUK134; *RH* reactive hyperemia; Small conductance calcium-activated potassium channel blocker: Apamin; *SMC* smooth muscle cells

↑ enhanced effect; ↓ reduced effect; –: the effect is not different

### Adenosine and adenosine receptor-mediated actions in pathological conditions

#### Hypertension

Hypertension is associated with structural and functional abnormalities in coronary microcirculation including coronary endothelial cell dysfunction, coronary microvascular remodeling and an impaired coronary flow reserve induced by adenosine observed in both human and animals [62, 105]. Arterial hypertension can lead to an increase in the vascular pericyte coverage, which is interestingly not accompanied by a gain in capillary density [127]. In addition, this cell type also undergoes a transformation into a more vascular smooth muscle cell like phenotype showing a more contractile property [127].

Both adenosine and the selective A_2A_R agonist produce concentration-dependent relaxation of coronary arteries isolated from control rats via activation of Kv_7_ channels but not hypertensive rats [43]. There is downregulation of A_3_R expression and the A_3_R-mediated coronary vasodilation in perfused hearts from spontaneously hypertensive rats [38]. In hypertensive swine, the transmural spatial density of microvessels is twice as much as in normotensive animals, and myocardial levels and expression of endothelium-derived growth factors, e.g., FGF (in vascular smooth muscle cells and myocytes) and VEGF (in endothelial cells) are significantly increased. Functionally, the increase in blood volume and myocardial blood flow in response to intravenous adenosine application was blunted in these animals [74]. It seems that activation of downstream potassium channels plays a role. In high-salt diet-induced hypertensive rats, application of nicorandil, an activator of K_ATP_ channels, restores NO synthase and attenuates enhanced VEGF and FGF gene expression resulting in coronary capillary and arteriolar growth [114]. These findings suggest that A_2A_R, A_3_R and potential downstream potassium channels might play a crucial role in coronary microvascular dysfunction in hypertension and potentially set a new strategy to pharmacological manipulation of coronary microvascular function by applying agonists stimulating these components (Table 2).

**Table 2:** Adenosine and adenosine-mediated coronary microvascular function in cardiometabolic disease

Disease	Species	Agent	Administration route	Receptor	Coronary effect	Reference
Hypertension	Human	Adenosine	Intracoronary infusion		Coronary flow reserve ↓	[105]
	Swine	Adenosine	Intravenous infusion		Myocardial microvascular funciton ↓	[74]
	Rat	Adenosine	Bolus injection in isolated arteries (diameter: ~ 200 μm)		Coronary relaxation ↓	[43]
	Rat	CGS21680	Bolus injection in isolated arteries (diameter: ~ 200 μm)	A_2A_R	Coronary relaxation ↓	[43]
	Rat	APNEA	Infusion in isolated hearts	A_3_AR	Coronary relaxation ↓	[38]
	Rat	Cl-IB-MECA	Infusion in isolated hearts	A_3_AR	Coronary relaxation ↓	[38]
Diabetes	Human with T2D	Adenosine	Intravenous infusion		Coronary flow reserve ↓	[48]
	Swine with MS	Adenosine	Intracoronary infusion	A_2B_R	Coronary blood flow -	[8]
	Swine with MS	2-CAD	Bolus injection in pressurized arterioles (diameter: 50–150 μm)	A_2B_R	Coronary reaxation -	[8]
	Rats with insulin resistance	Adenosine	Intravenous infusion		Myocardial microvascular funciton ↓	[102]
	Mouse with T1D	CGS21680	Infusion in isolated hearts	A_2A_R	Coronary flow ↑	[45]
	Mouse with T2D	Adenosine	Infusion in isolated hearts		Coronary flow ↓	[58]
Atherosclerosis	Human	Adenosine	Intracoronary infusion		Coronary flow reserve ↓	[72]
	Monkey	Adenosine	Bolus injection in pressurized arterioles (diameter 122–220 μm)		Coronary relaxation -	[84]
	Mouse	CGS21680	Infusion in isolated hearts	A_2A_R	Coronary flow ↑	[97]
	Mouse		Occlusion in isolated hearts	A_2A_R	Baseline flow and RH ↓	[120]
Ischemic heart disease	Swine	Up_4_A	Bolus injection in isolated arteries (diameter ~ 150 μm)	A_2B_R	Coronary relaxation ↓	[123]
	Dog	DPMA	Intravenous infusion	A_2A_R	Increase in coronary blood flow ↓	[16]
	Dog	Adenosine	Intracoronary infusion		Increase in coronary flow ↓	[103]

A_2A_R agonist: CGS21680; DPMA; A_2B_R agonist: 2-CAD; A_3_R agonist: APNEA, Cl-IB-MECA; MS: metabolic syndrome; T1D: type 2 diabetes; T2D: type 2 diabetes

↑ enhanced effect, ↓ reduced effect; – the effect is not different

#### Diabetes

Diabetes is an important risk factor for the development of cardiovascular disease including atherosclerosis and ischemic heart disease. The increased morbidity and mortality are significantly attributed to diabetes-induced microvascular dysfunction in the heart [119].

The coronary flow in hearts isolated from type 1 diabetic mice is observed to be significantly increased by the stimulation of the non-selective agonist of ARs and the selective A_2A_R agonist. In addition, in vivo injection of the A_2A_R agonist enhances the efficiency in increasing coronary flow in type 1 diabetic mice [45]. The functional observations are in accordance with the increased A_2A_R expression in coronary arteries as compared to non-diabetic control mice [45]. In contrast, the coronary flow in response to adenosine is significantly blunted in isolated hearts of type 2 diabetic Goto-Kakizaki (GK) rats as compared to age-matched control rats [58]. The impaired adenosine-induced coronary flow in GK rats can be restored by endothelial K_Ca2+_ opening [58]. In obese rats with insulin resistance, the coronary microvascular perfusion is impaired in response to adenosine infusion [102]. A similar clinical observation was found in a recent study where the adenosine-induced coronary flow reserve is blunted in type 2 diabetic patients without obstructive coronary artery disease [48] (Table 2). The different responses to adenosine stimulation may be due to different etiologies of diabetes, which warrants further investigations.

In a swine model with early-stage metabolic syndrome and hyperglycemia, despite both adenosine-induced increase in coronary blood flow in vivo and the adenosine analogue-mediated relaxation in isolated coronary arterioles did not differ from control swine [8], there was a shift from the A_2A_R-mediated coronary relaxation to enhanced A_2B_R-mediated coronary relaxation in swine with early-stage metabolic syndrome [8] (Table 2). However, the A_2B_R expression level was lower in coronary arterioles isolated from swine with metabolic syndrome. This may suggest that the sensitivity of A_2B_R upon stimulation by the adenosine analogue is increased thereby maintaining the coronary blood flow [8]. Moreover, the involvement of K_v_ channels in AR-mediated coronary relaxation was not affected by early-stage metabolic syndrome, whereas there was a reduced K_ATP_ channel function [8]. Activation of A_2A_R has been shown to be coupled to K_ATP_ channel to regulate coronary microcirculation [78, 86]. The reduced K_ATP_ function by early-stage metabolic syndrome can be affected by the shift of vasodilator A_2A_R.

#### Atherosclerosis

Atherosclerosis is generally predominant in large coronary arteries. However, long-term exposure to hypercholesterolemia can activate endothelial cells and thus induce leukocyte recruitment, oxidative stress and loss of pericytes in the microcirculation [12]. This alteration may lead to capillary rarefication due to a decrease in capillary surface area resulting in a dysfunctional downstream vessel system and a drastic decrease in overall capillary diameter [127]. Moreover, in atherosclerotic areas, relative anoxia, inflammation and oxidative stress promote release of angiogenic factors resulting in angiogenesis and vasculogenesis [57]. It has been estimated that local adenosine may mediate 50–70% of the angiogenic response to hypoxia/ischemia [2]. A_1_R, A_2B_R and A_3_R were involved in angiogenesis surroundings and downstream vessels of the atherosclerotic plaque, and A_1_R and A_2B_R were reported to promote endothelial progenitor cell homing to coronary microvascular endothelium for the genesis of capillary networks [77]. Regarding the action of adenosine on vascular tone regulation, the coronary microvascular responses to adenosine are not consistent in atherosclerosis. Low coronary flow reserve after intracoronary adenosine infusion was observed in patients with atherosclerosis risk [72]. Reactive hyperemia-induced increase in coronary flow was lower in female atherosclerotic mice, and the less increase in coronary flow was inhibited by the A_2A_R antagonist to a greater extent in atherosclerotic than control groups [120]. In contrast, an enhanced response in coronary flow to A_2A_R stimulation in hyperlipidemic/atherosclerotic mice was reported [97]. It has been suggested that upregulation of A_2A_R is a compensatory mechanism to maintain NO-dependent endothelial function as evaluated by coronary vasodilation in a mouse model of atherosclerosis [120]. One study indicates that responses of isolated coronary arterioles to adenosine are identical in atherosclerotic and control monkeys [84] (Table 2). The experimental evidence may suggest that the diagnosis of coronary artery disease in patients using adenosine as a stimulator can be underestimated.

#### Ischemic heart disease

Among complex pathophysiological components, e.g. obstructive coronary atherosclerosis, more and more evidence has shown that coronary microvascular dysfunction significantly plays a role in the etiology of ischemic heart disease [54, 70, 94]. On the other hand, the coronary vasculature itself is also a victim of ischemia–reperfusion injury and myocardial infarction [30, 37]. The majority of experimental and clinical studies have focused on the effects of adenosine more on cardiomyocytes as compared to the coronary vasculature, as dissecting the effects of adenosine or AR activation on the coronary microcirculation from cardiomyocytes is challenging, given the causal relationship between injuries to the coronary vasculature and cardiomyocytes following the myocardial infarction.

Existing data demonstrated that there seems to be a reduced sensitivity to adenosine in the coronary microvasculature in ischemic heart [103, 128]. the A_2_R agonist-induced coronary vasodilation was attenuated by the ischemia–reperfusion in anesthetized dogs [16]. The AR-, likely A_2B_R-mediated relaxation to the novel dinucleotide Up_4_A in isolated coronary small arteries was found to be blunted in swine with myocardial infarction [123] (Table 2). More studies are needed to further elucidate the specific AR involvement in coronary microvascular function following myocardial infarction and how alteration of AR sensitivity is associated with ischemic heart disease.

### Perspective on indirect adenosine modulation as therapeutic strategy

Adenosine and AR modulations may serve as therapeutic strategy in cardiovascular medicine in two manners. First, both endogenous and exogenous adenosine and adenosine-activated ARs per se have been evaluated in various preclinical and clinical settings. However, the effect of adenosine and AR modulation in myocardial injury and heart failure has shown inconsistent effects on cardiac function and myocardial perfusion. The exact mechanisms are not readily explained, but one possibility may rely on where the modulation takes place. For instance, endogenous generation of interstitial, but not venous adenosine, is critical to protect myocardium against infarction [83, 87], which could be induced by ischemic preconditioning, but not coronary microembolization [87]. Moreover, none of the pharmacological tools targeting ARs that entered clinical trials have emerged as drug candidates due to lower efficacy, kinetics issues or adverse events reported. Better rational design and development of other agonists and antagonists may lead to successful clinical drug candidates in the future. Readers are referred to several review articles on this topic for more details [14, 61, 111]. Second, other drugs can initiate secondary effects through generation of adenosine and activation of AR-mediated signaling. It is important to note that AR-mediated actions may affect both coronary vasculature and cardiomyocytes, making it difficult to separate vascular effect from cardio-protection. This section focuses on the discussion of the indirect adenosine modulation for a potential therapeutic strategy.

An indirect, but clinically important, effect on AR-mediated signaling was recently postulated for ticagrelor [17]. Ticagrelor is the P2Y_12_R antagonist primarily targeting platelets and its application is clinically wellestablished to prevent thromboembolic complications after acute coronary syndrome [107]. Of note, ticagrelor can induce substantial amount of ATP release from erythrocytes via anion channels and target the ENT1 transporter in erythrocytes which inhibits adenosine uptake by erythrocytes [66, 110]. Together with adenosine degraded from ATP in this pathway, ticagrelor, by targeting erythrocytes, leads to increases in circulating adenosine levels [101]. Considering the beneficial effects of adenosine on cardiovascular function [110], ticagrelor could have pleiotropic effects beyond its platelet inhibitory effects, as treatment with ticagrelor reduced major cardiovascular adverse events (MACE) compared to clopidogrel, another P2Y_12_R antagonist that does not have impact on erythrocytes for purinergic activation, in patients with acute coronary syndrome [13]. Indeed, increased adenosine concentrations by ticagrelor reduced anti-inflammatory responses, improved vascular function and attenuated ischemia–reperfusion injury [13]. Moreover, ticagrelor significantly enhanced adenosine-increased coronary blood flow in human and adenosine-mediated hyperemia in dogs [101, 112]. Of note, a clinical study evaluating the effects of ticagrelor in stable multivessel ischemic heart disease is ongoing [17]. However, how much adenosine-mediated secondary effect of ticagrelor contributes to overall cardiovascular outcomes remains unclear.

In addition to ticagrelor, magnesium has been applied in patients for possible treatment of acute myocardial infarction [113]. Evidence has shown that the beneficial effect of magnesium in an animal model of myocardial infarction on the infarct size is by adenosine through enhancement of 5′-nucleosidase activity [55]. It is of interest to monitor the effect of magnesium on the coronary microvascular function. Further studies are required to better elucidate the extent to which enhanced adenosine responses contribute to the clinical profile of those compounds. More studies aiming at pinpointing ARs and manipulating receptor sensitivity in coronary microvasculature are also needed to evaluate the possible therapeutic potential.

## Conclusions and perspectives

Adenosine is an endogenous purine nucleoside that functions as an extracellular signaling molecule via activation of ARs. Adenosine and adenosine-mediated ARs play a significant role in the regulation of coronary microcirculation in certain conditions in physiology and pathophysiology. Adenosine mediates coronary microvascular tone and reactive hyperemia mainly through A_2A_R activation on both endothelial and smooth muscle cells and also via interaction with other ARs. ARs further activate the downstream effectors including H_2_O_2_, K_ATP_, K_V_ and K_Ca2+_ channels leading to coronary vasodilation.

Adenosine-mediated AR activation also plays a role in several cardiovascular diseases. Downregulation of A_2A_R, A_3_R and potential downstream potassium channels play a crucial role in coronary dysfunction in hypertension. A_1_R, A_2B_R and A_3_R are thought to be involved in the angiogenesis and microvascular growth in coronary atherosclerosis. The coronary microvascular responses to adenosine are not consistent in atherosclerosis, which may underestimate diagnosis of coronary artery disease using adenosine as a stimulator. There is a decreased A_2_R sensitivity in coronary microcirculation after ischemia–reperfusion and myocardial infarction. The adenosine effect on coronary flow regulation in diabetes is not consistent and may depend on the etiology of diabetes. More studies are needed to evaluate the adenosine and AR modulation for the treatment. Indirect modulation of adenosine by a compound like ticagrelor may be of potential for the improvement of coronary microvascular function in certain cardiovascular disorders.

Collectively, there is a complexity of adenosine and AR-mediated effects in coronary microcirculation. Many aspects are still not fully understood due to a number of discrepant observations. The discrepancy arises from (1) endogenous adenosine vs. exogenous adenosine effects and adenosine concentration vs. AR sensitivity, (2) different conditions/stimuli (basal condition, ischemia, hypoxia, exercise/pacing and diseases) and (3) differences in AR expression and distribution in different microvascular segments of different species. Better understanding of these aspects will help with elucidation of the role of adenosine and AR in the regulation of coronary microcirculation and development of novel therapeutic strategies.
